# Is the Therapeutic Mechanism of Repetitive Transcranial Magnetic Stimulation in Cognitive Dysfunctions of Depression Related to the Neuroinflammatory Processes in Depression?

**DOI:** 10.3389/fpsyt.2022.834425

**Published:** 2022-02-24

**Authors:** Hiroshi Tateishi, Yoshito Mizoguchi, Akira Monji

**Affiliations:** Department of Psychiatry, Faculty of Medicine, Saga University, Saga, Japan

**Keywords:** depression, repetitive transcranial magnetic stimulation, cognitive dysfunction, neuroinflammation, tryptophan metabolites, white matter integrity

## Abstract

The lifetime prevalence of depression is reported to be >10%, and it is an important illness that causes various disabilities over a long period of life. Neuroinflammation process is often reported to be closely linked to the pathophysiology of depression. Approximately one-third of depression is known to be treatment-resistant depression (TRD), in which the symptoms are refractory to adequate treatment. Cognitive dysfunction is one of the most important symptoms of depression that impedes the rehabilitation of patients with depression. Repetitive transcranial magnetic stimulation (rTMS) is a minimally invasive and effective treatment for TRD and is also known to be effective in cognitive dysfunction in depression. Since the details of the therapeutic mechanism of rTMS are still unknown, we have been conducting studies to clarify the therapeutic mechanism of rTMS, especially focusing on cognitive dysfunction in depression. In the present review, we present our latest results and discuss them from the standpoint of the neuroinflammation hypothesis of depression, while citing relevant literature.

## Introduction

The lifetime prevalence of depression is 21%. It is an important illness that causes various disorders over a long period of time (1). Approximately one-third of patients are known to have treatment-resistant depression (TRD), in which the symptoms are refractory to adequate treatment (2).

**Figure 1:**
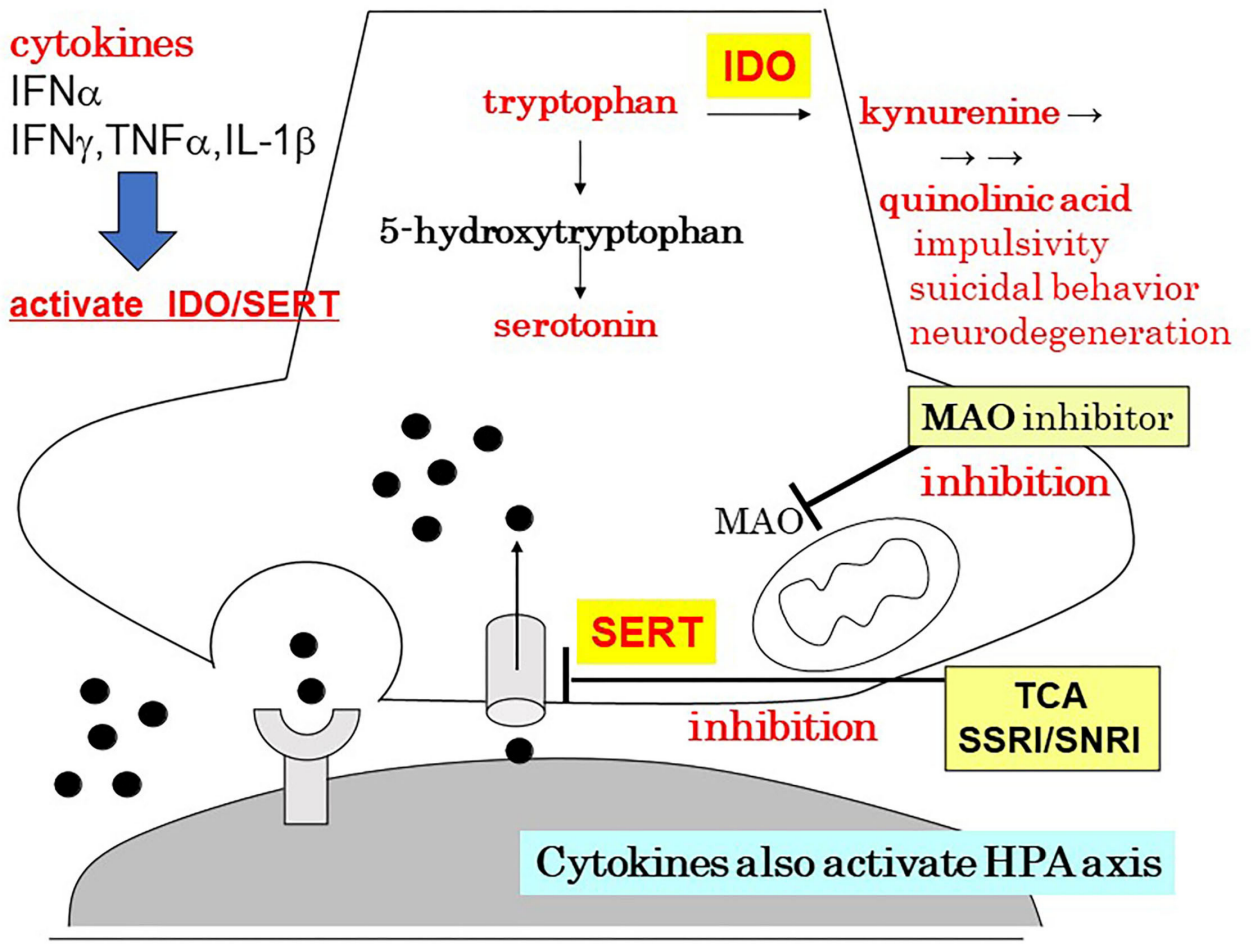
The neuroinflammation hypothesis of depression. Author's modification from Monji (6). HPA, hypothalamic–pituitary–adrenal axis; IDO, indoleamine 2,3-dioxygenas; IFN, interferon; IL, interleukin; MAO, monoamine oxidase; SERT, serotonin transporter; SNRI, serotonin/noradrenaline reuptake inhibitor; SSRI, serotonin reuptake inhibitor; TCA, tricyclic antidepressant; TNF, tumor necrosis factor.

Patients with major depression have been found to exhibit increased levels of peripheral blood inflammatory biomarkers including inflammatory cytokines (3). The association between inflammation and major depression has been one of the leading hypotheses for over a decade (3). Recent studies have also reported that the inflammatory process is involved in the pathophysiology of depression (4, 5). A schema of the neuroinflammation hypothesis of depression is shown in Figure 1 (6). Recently, TRD was shown to be associated with increased inflammatory processes (7, 8). The concentrations of many inflammatory proteins were higher in patients with TRD than in the control group, and poorer responses to treatment were associated with elevated levels of interleukin (IL)-6 and 8, tumor necrosis factor (TNF), C-reactive protein (CRP), and macrophage inflammatory protein-1 (7). There is a significant relationship between higher plasma levels of inflammatory cytokines, including IL-6, and the number of failed treatment trials (8). Patients who failed three or more trials in the current episode revealed significantly higher plasma levels of inflammatory cytokines, including IL-6, compared to patients with less than one trial by *post-hoc* pairwise comparisons with correction for multiple testing (8). More treatment failures were also associated with high-sensitivity CRP only in models with body mass index excluded (8).

A meta-analysis investigated changes in inflammatory cytokines due to selective serotonin reuptake inhibitor (SSRI) treatment in patients with depression (9). This meta-analysis included 22 eligible studies of 827 patients with major depressive disorder (MDD): seven studies for IL-1β, 15 for IL-6, 11 for TNF-α, six for IL-4, and four for interferon-γ. The pooled effect estimate indicated that the levels of the pro-inflammatory markers IL-1β, IL-6, and TNF-α were reduced by SSRI treatment. However, there was a high degree of heterogeneity among the studies included in this meta-analysis.

Antidepressants, psychostimulants, and non-pharmacological therapies improve cognitive dysfunction in patients with MDD. However, no reports have investigated the relationship between inflammatory cytokines and their therapeutic effects. Anti-cytokine drugs such as tocilizumab (targeting IL-6), infliximab, etanercept, and adalimumab (all targeting TNF-α) significantly improve depressive symptoms in humans (10–12), but little is known about the ameliorating effect of anti-cytokine treatment on cognitive dysfunction.

Cognitive dysfunction is closely associated with disorders experienced by many patients with depression (13). Cognitive dysfunction is persistent and observable in many patients with depression from the initial onset of depression to remission (14). Cognitive deficits affect several areas of social functioning, such as employment, social life, family life, and family responsibilities (13). More favorable outcomes, including depression-related psychosocial disorders, are associated with a shorter duration of untreated depression (15). In several neuropsychological studies, cognitive dysfunction has been found to involve a wide range of cognitive areas of depression, including executive function. Executive dysfunction associated with frontal lobe dysfunction has been reported to be prominent in patients with depression (16). Cytokines may affect cognition via various mechanisms. The roles of IL-1, IL-6, and TNF-α have been highlighted in most studies investigating the mechanisms by which cytokines are involved in cognitive dysfunction (17). Recent reports have shown that enhanced inflammatory processes reduce functional brain connectivity that is closely associated with cognitive dysfunction (18–20).

Repetitive transcranial magnetic stimulation (rTMS) is a minimally invasive and effective treatment for TRD and is known to be effective in treating cognitive dysfunction in depression. A systematic review and meta-analysis have shown that rTMS treatment targeting the prefrontal cortex in patients with MDD may moderately enhance cognitive function in set-shifting ability, visual scanning, and psychomotor speed (21). Tong et al. showed that rTMS might improve executive function in patients with MDD (22). Repetitive transcranial magnetic stimulation is considered promising and valuable for improving cognitive dysfunction in TRD (23).

Since the details of the therapeutic mechanism of rTMS are still unknown, we have been conducting studies to clarify the therapeutic mechanism of rTMS, especially focusing on the effect of rTMS on cognitive dysfunction in depression. In the present review, we present our latest results and discuss them from the standpoint of the neuroinflammation hypothesis of depression, while citing relevant literature.

## rTMS and Neuroinflammation

In MDD, the release of increased levels of pro-inflammatory cytokines and hormones in the plasma and indicators of oxidative stress have been identified as consequences of the activation of inflammatory pathways in the brain (4). The major supply of IL-1β in the central nervous system is provided by microglia. Many studies have reported that microglia are closely associated with the pathophysiology of depression in animal models (24). Administration of lipopolysaccharide stimulates the expression of IL-1β mRNA primarily in the cortical regions (the frontal and parietal cortex), hypothalamus, hippocampus, pituitary gland, thalamic nuclei, and cerebellum of rat (25) and mouse brains (26). In animal models, IL-1β injection causes hippocampal-dependent learning, memory impairment, and long-term potentiation impairment (27). In rodent models, IL-1β has been shown to have stress-induced anhedonic and anti-neurogenic effects (28).

It has been reported that rTMS decreases serum IL-1β and TNF-α levels in elderly patients with TRD (29).

**Figure 2:**
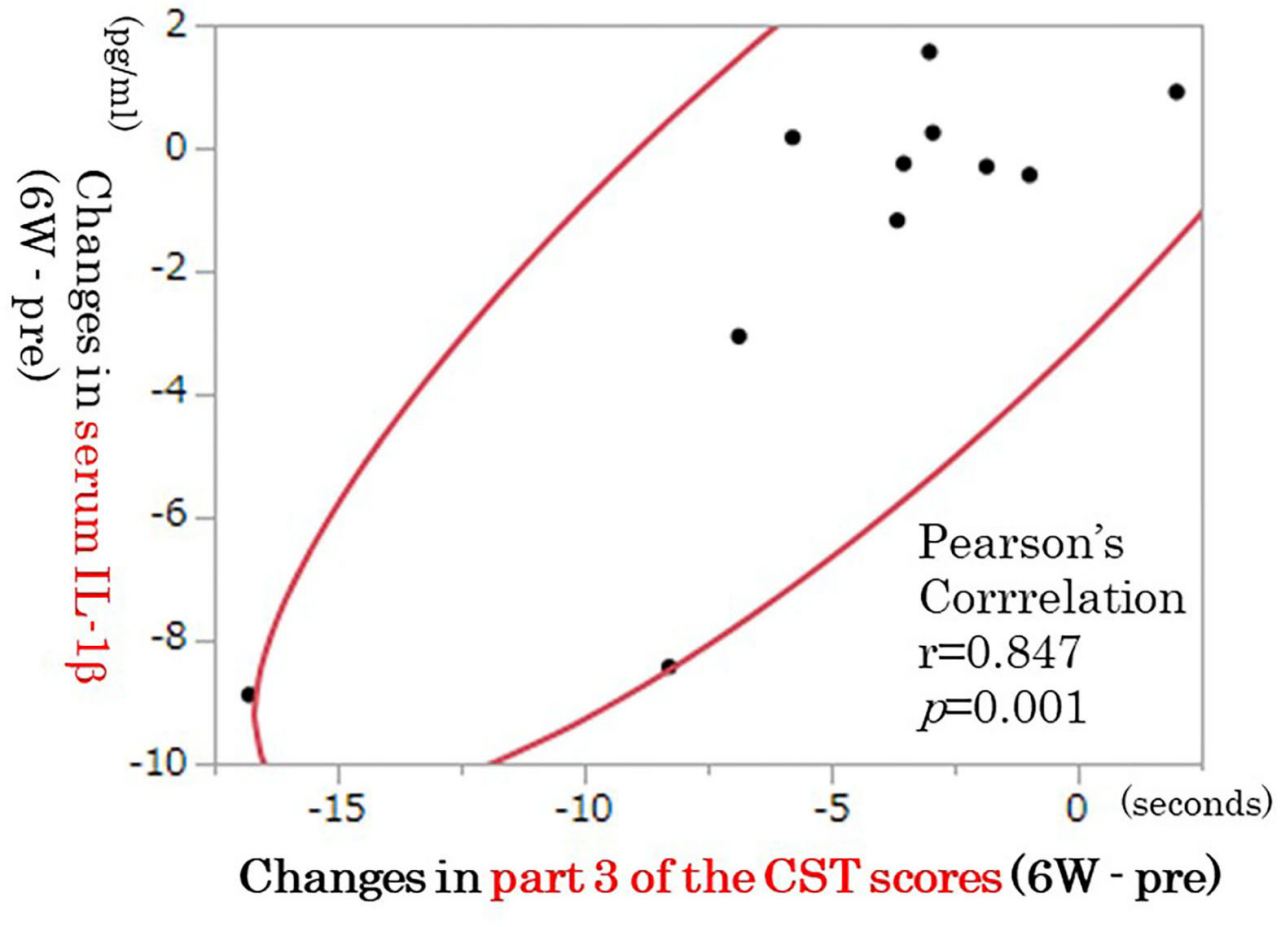
Correlation between changes in IL-1β and part 3 of the CST scores (*n* = 11). The decrease in IL-1β was correlated with improvement in part 3 of the CST scores (*p* = 0.001, *n* = 11). IL, interleukin; CST, color Stroop test.

We have demonstrated that 6 weeks of rTMS treatment significantly improved the Hamilton Depressive Rating Scale (HAM-D), Beck Depression Inventory, total errors of the Wisconsin Card Sorting Test (WCST), category of the Word Fluency Test (WFT), and part 3 of the Color Stroop Test (CST) scores (30). Although serum IL-1β, IL-6, and TNF-α levels were not significantly changed by rTMS, serum IL-1β tended to decrease (30). This study suggested that rTMS tended to decrease serum IL-1β independently of improvement in depressive symptoms and that rTMS improved partial cognitive dysfunction independently of improvement in depressive symptoms (30). Decreased serum IL-1β by rTMS was correlated with partial improvement in cognitive dysfunction (CST score part 3) (Figure 2) (30). Changes in various cytokines have been associated with cognitive dysfunction observed in depression in human and animal models (17). The ameliorating effect of rTMS on cognitive dysfunction in patients with TRD may be related to changes in IL-1β, but further studies on the involvement of inflammatory cytokines other than IL-1β are needed in the future.

## rTMS and Tryptophan Metabolites

**Figure 3:**
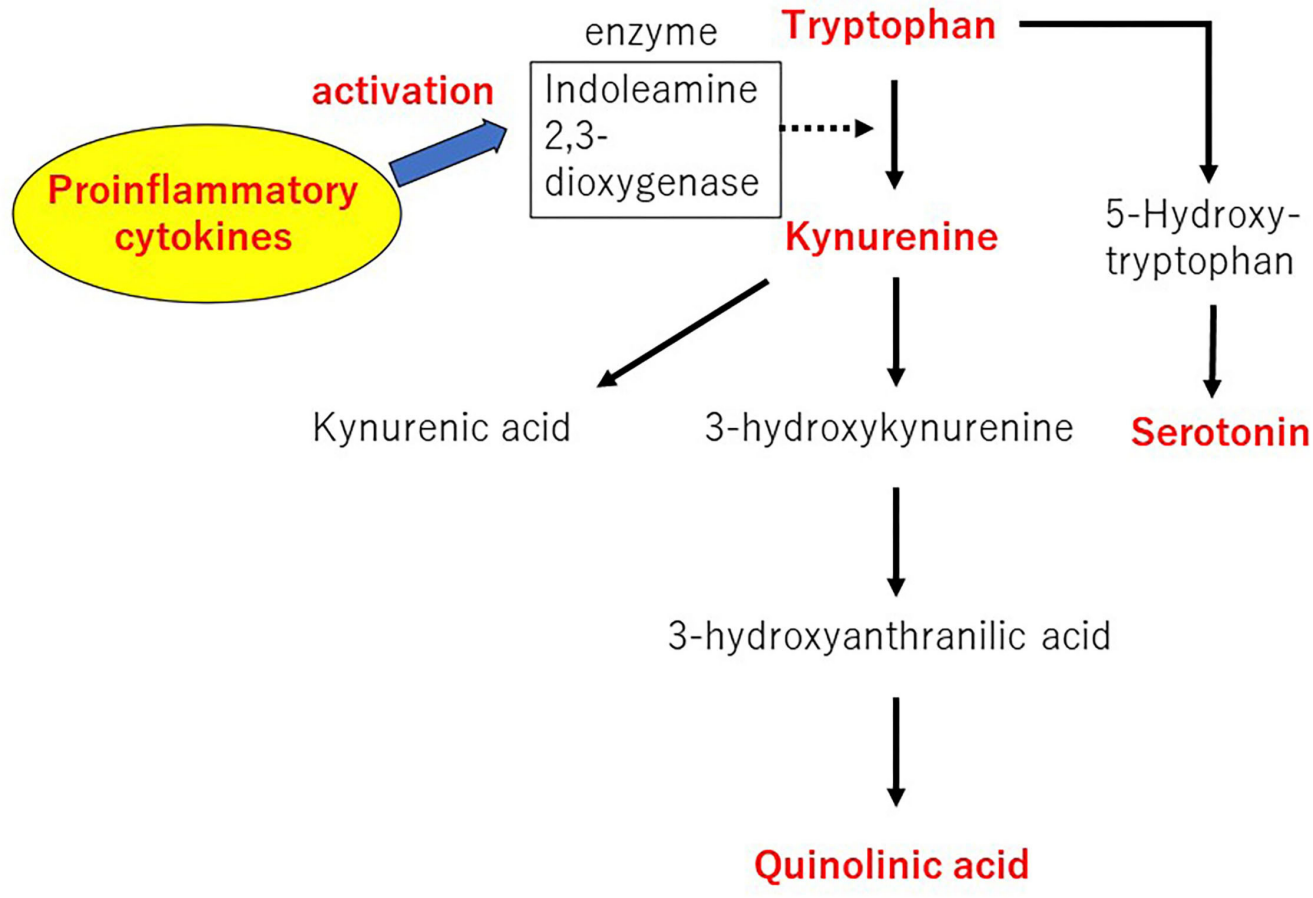
The kynurenine pathway of tryptophan metabolism. Author's modification from Tateishi et al. (31).

Tryptophan (TRP) is metabolized to several bioactive molecules, the most famous of which is serotonin. However, only a small portion of the TRP is converted to serotonin. Kynurenine (KYN) and its degradation products are metabolites of 95% or more TRP via the kynurenine pathway (KP). These are collectively known as KYNs (31). The KP scheme for TRP metabolism is shown in Figure 3 (31).

Numerous studies have reported changes in TRP metabolite levels in patients with depression. Patients with MDD have significantly lower plasma TRP and KYN levels than the controls (32). The higher concentration of plasma KYN to KYNA and KYN/TRP to KYNA/KYN ratios during pregnancy and lower concentration of plasma 3-hydroxyanthranylic acid during the postpartum period have been shown to be closely associated with postpartum depressive symptoms (33). Profound changes in TRP metabolism have been reported in patients with late-onset depression, whereas low TRP levels and changes in KYN metabolism have been reported in patients with early-onset depression (34). It has been suggested that the increased risk of depression observed after interferon-α administration in patients with chronic medical illness is likely to be mediated by the KP (35). Erabi et al. demonstrated that KYN and KYNA levels were significantly and negatively associated with reduced HAM-D in 62 patients with MDD treated with escitalopram for approximately 6 weeks (36). Patients with MDD have lower KYNA levels, in which only one of the 73 metabolites is detected, and lower KYNA levels are associated with better treatment responsiveness to escitalopram (36). Patients with MDD, especially the less personality-biased group, showed significantly lower levels of plasma metabolites in the TRP pathway, including TRP, serotonin, and KYN (37). Antidepressant treatment may affect plasma levels of KYN-related metabolites. Antidepressant treatment has been reported to be likely to normalize KYN pathway dysfunction both in preclinical and clinical trials (38). Eskelund et al. found that vortioxetine reduced quinolinic acid levels in many brain regions in both genetic rat models and mouse models, with increased inflammation-induced depression-like behavior (39).

Electroconvulsive therapy (ECT) is an effective treatment for TRD, and KP is involved in the therapeutic mechanism of ECT. Several recent studies have investigated the changes in KYN metabolism during ECT in patients with MDD. Guloksuz et al. showed that treatment with ECT increases KYNA levels and KYN/TRP, KYNA/KYN, and KYNA/3-hydroxyquinurenin ratios during ECT and up to 6 weeks after the last ECT (40). In a study by Schwieler et al. ECT significantly reduced the plasma levels of TRP, KYN, and quinolinic acid, but did not change the plasma levels of KYNA (41). Electroconvulsive therapy treatment significantly reduces the quinolinic acid/KYNA ratio (41). In a study by Ryan et al. an increase in KYN, 3-hydrochianthranilic acid, 3-hydroxyquinurenin, quinolinic acid, and KYN/TRP ratios correlated with improved mood scores after ECT in a subgroup of patients with MDD (42). Aarsland et al. found that ECT treatment increased the levels of neopterin, an inflammatory marker, and 3-hydrochianthranilic acid and picolinic acid, putative neuroprotective KYNs in patients with MDD (43).

There have been no metabolomic studies of TRP metabolites for rTMS treatment in patients with TRD. We demonstrated that plasma TRP levels were significantly increased and plasma serotonin levels were significantly decreased in TRD patients after 6 weeks of rTMS treatment, but plasma KYN and kynurenic acid levels and KYN/TRP ratios were not altered (31). The increase in melatonin levels and improvement in categories achieved of the WCST and deterioration in part 1 of the CST scores showed significant correlations (31). Although rTMS increased serum TRP levels, the extent to which rTMS improves cognitive function through changes in TRP metabolites requires further study with an increased sample size.

Elevated peripheral pro-inflammatory cytokines, such as IL-1β, are observed in some subgroups of patients with MDD (44, 45). Inflammatory cytokines such as IL-1β can ultimately increase KYN formation through the activation of indoleamine 2,3-dioxygenase, a TRP-degrading enzyme (46). Kynurenines play an important role not only in immunomodulation but also in the pathology of various diseases including depression (47, 48). The effect of rTMS on IL-1β may also affect KP. In future studies, inflammatory cytokines involved in KP should be investigated.

## rTMS and White Matter Integrity

The relationship between peripheral pro-inflammatory markers, such as IL-1β, IL-6, TNF-α, and CRP, in patients with MDD and functional and structural neuroimaging markers in magnetic resonance imaging (MRI) is being increasingly investigated (49). Functional MRI studies have shown a correlation between blood levels of pro-inflammatory markers and abnormal activation patterns and functional connectivity changes in neural circuits involved in cognitive control, emotional regulation, and reward processing in depression (49). Structural MRI studies have shown correlations between blood levels of pro-inflammatory markers and cortical thinning, decreased cortical gray matter and subcortical volume, and decreased integrity of the white matter tract in neural circuits associated with patients with MDD (49).

To date, only a few studies have investigated the association between rTMS-induced changes in the white matter microstructure and therapeutic response (50, 51). Kozel et al. reported that active stimulation of rTMS did not cause harmful damage to the white matter compared to the sham stimulation group (50). Peng et al. reported that rTMS significantly reduced fractional anisotropy (FA) in the left middle frontal gyrus in patients with TRD using a voxel-based analytical method (51). Active rTMS treatment significantly improved this reduction of FA, but sham rTMS treatment did not.

We suggest that rTMS significantly improves depressive symptoms and some cognitive dysfunction (category in WFT and part 3 of CST scores) in patients with TRD (52). Repetitive transcranial magnetic stimulation treatment improved the category in WFT and part 3 of the CST scores independently of the improvement of depressive symptoms (52). We demonstrated that the amelioration of cognitive dysfunction induced by rTMS is not associated with increased white matter integrity (FA value) induced by rTMS in patients with TRD (52). Although rTMS affects the FA values of the cerebral white matter in patients with depression, further research is needed on how it is related to the improvement of cognitive function with a larger sample size.

Inflammatory cytokines, such as IL-1β, are histologically damaging to oligodendrocytes and can lead to histological changes such as white matter lesions found in patients with depression (53, 54). The effect of rTMS on IL-1β may affect white matter integrity, and further studies are needed to examine the association between inflammatory cytokines, including IL-1β, and cerebral white matter integrity. Cognitive dysfunction in MDD may be associated with aberrant functional connectivity in default mode network and executive control network using resting-state fMRI (55). Meta-analysis revealed that clinical response to rTMS treatment, ECT, transcutaneous vagus nerve stimulation, cognitive behavioral therapy, and pharmacotherapy could be predicted by baseline default mode network connectivity in patients with depression (56). The rTMS treatment had larger effect size compared to other treatment strategies. It is possible that the cognitive function improving effect of rTMS directly or indirectly affects the brain network.

## Conclusions and Future Perspectives

Our latest research results and relevant literature suggest that the therapeutic mechanism of rTMS in cognitive dysfunction of depression could be related to neuroinflammatory processes mainly mediated by the pro-inflammatory cytokine IL-1β. We make assertions based on correlational data more than clearly defined causative interactions, which we believe warrants further study. Electroconvulsive therapy alters various cytokines in patients with MDD and is associated with changes of affective states such as depressed mood (57). Comparison of rTMS and ECT with respect to the therapeutic mechanism is necessary for future research. Whether the therapeutic mechanism of rTMS based on the neuroinflammatory hypothesis is essential not only for MDD but also for other psychiatric disorders such as obsessive-compulsive disorder and schizophrenia, further research is needed. Future studies on the synergistic effects of neuromodulation such as rTMS and pharmacologic approaches such as SSRIs and anti-cytokine drugs are useful.

## Author Contributions

HT and AM drafted and revised the manuscript, respectively. YM conducted a literature review under the supervision of HT and AM. All authors have approved the final submitted manuscript.

## Funding

This research was supported in part by the Grants-in-Aid for Scientific Research from the Japan Society for the Promotion of Science [JSPS KAKENHI, Grant Number JP17K10307 (to HT)].

## Conflict of Interest

The authors declare that the research was conducted in the absence of any commercial or financial relationships that could be construed as a potential conflict of interest.

## Publisher's Note

All claims expressed in this article are solely those of the authors and do not necessarily represent those of their affiliated organizations, or those of the publisher, the editors and the reviewers. Any product that may be evaluated in this article, or claim that may be made by its manufacturer, is not guaranteed or endorsed by the publisher.
